# People’s willingness and determinants to use selected tele-consultation public health services in Mozambique

**DOI:** 10.1186/s12889-021-10709-9

**Published:** 2021-05-19

**Authors:** Germano Vera Cruz, Priscilla S. Dlamini

**Affiliations:** 1 Department of Psychology, Faculty of Education, Eduardo Mondlane University, Campus Universitário Principal, Av. Julius Nyerere, N°3453, CP257 Maputo, Mozambique; 2 Department of General Nursing, University of Eswatini, Kwaluseni, M201 Eswatini

**Keywords:** Sub-Saharan Africa, Mozambique, Telemedicine, Tele-consultation

## Abstract

**Background:**

To achieve universal health coverage by 2030, sub-Saharan African countries are planning to develop large scale tele-consultation public health services. However, there is a lack of knowledge regarding the level of peoples’ willingness to use this kind of tele-health services. To address this gap and inform policymakers, the present study aims at accessing the Mozambican people’s willingness to use tele-consultation public health services and the determinants associate to their willingness.

**Methods:**

A total of 403 adults participated in the study. The material consisted of 32 vignettes (scenarios) describing realistic *health problem* situations in which an individual was proposed to use a tele-consultation public health service, varying as a function of five factors: consultation category, health problem category, health problem severity, physician category, and the consultation price. For each health problem situation presented in the vignettes, the participants were asked to rate their willingness to use the proposed tele-consultation service on an 11-point scale. A cluster analysis using the K-means procedure was applied to the quantitative raw data to capture the participants’ different perspectives. ANOVA, x^2^ and t-test analyses were also conducted to examine the effects of the different health problem situations and the sociodemographic characteristics on the participant ratings.

**Results:**

Five different perspectives (clusters) were found: *never-willing* (15% of the sample), *severity* (26%), *consultation-category* (22%), *undecided* (16%), and *price-severity* (21%). These perspectives were associated with participants’ sociodemographic characteristics.

**Conclusion:**

According to the main results, it seems that the majority of the participants (69%) were highly willing to use tele-consultation public health services in the case of mild illness, cheaper prices and follow-up consultation. In addition, the participants’ willingness was significantly affected by some of the participants’ sociodemographic characteristics.

**Supplementary Information:**

The online version contains supplementary material available at 10.1186/s12889-021-10709-9.

## Background

In the majority of the sub-Saharan African countries the coverage rates for public health services are extremely low. They have the greatest shortage of health professionals in the world [1]. In many of these countries, the access to health services and health professional care is only possible after long hours of travel or waiting in long queues in the existing health facilities [2]. For instance, the sub-Saharan African countries have the lowest Universal Health Coverage Index (UHCI = 42) and lowest access to basic health care services (25%). Amongst all the sub-Saharan African countries, this index is higher in South Africa (UHCI = 67) and the lowest in Somalia (UHCI = 22) [1]. In the referred sub-continent region, physician-to-population ratio is estimated at 0.2%. This ratio is higher in Sudan (3.1%) and lower in Somalia (0.0%) [3].

The poor level of access to health services in sub-Saharan Africa leads to poor health indices [2]. For example, communicable diseases such as HIV/AIDS, tuberculosis, malaria, and others have been endemic public health problem for many decades, and this high prevalence of communicable diseases has now been complicated by the current high prevalence of non-communicable diseases such as hypertension, diabetes, cancer and severe psychological/psychiatric disorders [2].

To “build a healthier world for all” and achieve universal health coverage by 2030, the representatives of States and Governments assembled in Tokyo (2017) and in New York (2019) to sign a declaration reaffirming their intentions to scale up the global effort and investments on sustainable public healthcare systems [4, 5]. Following this commitment, the sub-Saharan leaders decided to improve levels of access and utilisation of healthcare services by developing innovative solutions based on telemedicine, together with measures such as investment in traditional health infrastructures and personal training [4, 6].

The World Health Organization (WHO) define telemedicine (including tele-health, e-health, m-health and tele-consultation) as “the delivery of health care services, where distance is a critical factor, using information communication technologies (ICT) for exchange of valid information for diagnosis, treatment, prevention of diseases, injuries, research, evaluation, and for continuing education of health care providers, all in the interest of advancing the health of individuals and their communities” [7]. In general, applications of telemedicine range from the general use of the telephone for consultations between patients and clinicians (tele-consultation) to remote specific medical procedures such as tele-cardiology, tele-radiology, tele-dermatology, tele-surgery, etc. [9, 10]. In particular, telemedicine uses tools such as the internet, email, phone, website and videoconference applications to provide health services to patients (e.g., tele-consultations, remote patient monitoring, medication adherence), to health professionals (e.g., professional training, remote physician-to-physician collaboration), and to populations at risk (e.g., educational and prevention campaigns) [9, 10].

Focusing on tele-consultation, it is defined as the remote medical information exchange between a health care professional and a client/patient (e.g., a doctor and a patient) to improve the health of the patient [7, 8]. This information exchange can be carried out through two-way video conferencing, smart phones or other wireless tools.

While in sub-Saharan Africa telemedicine is still at an early stage, at present, a variety of tele-consultation pilot projects are being developed, and in the near future, many countries are planning to offer permanent tele-consultation health services to their population [9–11]. For instance, Nigeria, the most populated country in Africa, has been conducting tele-consultation pilot projects, linking medical experts with patients in remote areas [12, 13]. In recent years, the South African Department of Health has been using tele-consultation services in areas with a shortage of specialist health professionals [14, 15]. In addition to the projects mentioned above, recent studies reported that there are dozens tele-consultations pilot programmes operating in African countries [10], and that the emergence of Coronavirus Disease 2019 (COVID-19) pandemic may accelerate the uptake of tele-consultation public health services in the region [16].

In short, the barriers that could be addressed using tele-consultation are linked to the lack of physical infrastructures, costs, shortage of skilled health professionals and other resources in remote areas [17]. Indeed, the majority of the pilot projects developed across the sub-continent have shown that the rapid progress of cellular network coverage and mobile internet connectivity in the region^1^ [18] have the potential to extend considerably the accessibility of the healthcare services. This technology can enable public health services to reach the population in need more quickly and at a lower cost than through the development of the physical infrastructure. These projects have also showed that the use of tele-consultation reduce hospital burden, save the patients precious time, and increase the quality of healthcare [19]. Health personnel, who are generally concentrated in certain regions or in urban areas, can, through tele-consultations, provide a health service to populations in areas without health personnel while saving resources and travel time [19–21]. In addition, there is evidence of the cost effectiveness of tele-consultation for the patients [20, 21]. As matter of fact, patients can make cost savings because they do not need to travel long distances to receive their treatment, and their medical diagnosis can also be provided through online consultation or by video conferencing [20, 21].

However, to have the desired effect, public health tele-consultation services must address very specific and evidence-based health “needs” of each country and in each group category, as well as the social issues related to those needs [9, 10, 19, 22]. For instance, there is little published knowledge about the level of peoples’ willingness (both among the general and specific population groups) for tele-consultation in sub-Saharan African countries [23]. To what extent is the population concerned willing to use tele-consultation health care delivery services and which factors may determine their willingness? Attempts to find answers to this question are important since review studies have shown that, in sub-Saharan Africa, a significative number of “necessary” health programmes fail due to the fact that day were designed based on poorly vetted assumptions and without taking into account the social and cultural factors that may influence the population’s willingness to use them [24, 25]. In addition, the scarcity of resources in these African countries necessitates that health interventions should be acceptable to the general population to ensure the use of the services. Above all, this kind of study might help policy decisions on tele-consultation services scale-up, specificities and deployment.

From the search made in several databases and repositories (PubMed/Medline, Scorpus, Google Scholar, ResearchGate) based on the following keywords “tele-consultation”, “telemedicine”, “health services”, “sub-Saharan Africa”, one study related to the current study was found. The referred study, conducted in Nigeria in 2017, established that 48.7% of the respondents were willing to pay for tele-consultation [26]. The respondents’ socioeconomic status was the main statistically significant variable explaining their willingness to pay for tele-consultation [26]. As the aim of this study was to examine the consumers’ willingness to pay for tele-consultation services, it did not address questions on the willingness to use tele-consultation in general (whether it is paid for or not). The study also did not explore the determinant factors of that willingness.

Several research articles reporting the acceptability or the people’s willingness to use tele-consultation health services in other regions of the globe were found; however, all of them concerned very specific areas of medicine. For instance, a study conducted in the USA established that more than half of the patients surveyed at a tertiary care centre were willing to consider using tele-dermatology services [27]. A systematic review study of the acceptability of online and mobile phone tele-consultation for severe mental health problems showed that while 64% of participants (from USA, Canada, United Kingdom and India) were willing to have clinicians contact them via social media during symptom emergence, only 14 to 41% of them showed interest in mobile phone check-ins to inform health care providers about symptoms; significant relationships were reported between interest in tele-consultation and age, sex, race and employment status [28]. A study carried out in China reported that 68% of the participants showed interest in using an m-Health solution to manage their cardiovascular diseases; patients who were more willing to use m-Health solutions to manage their diseases were younger, more educated, possessed higher skills regarding mobile or internet device use, and more frequently searched for health information on the internet [29]. Finally, a systematic review of 45 studies on online consultation from the patient’s home identified 17 acceptability facilitators and eight acceptability barriers [30]. Among the facilitators, the most commonly cited are cost savings, reducing travel time, rapid accessibility to health services when in need; among the barriers, the most commonly referred are the impossibility to physically examine by the physician, preference to face-to-face interaction and unfamiliarity with the technology used [30].

### The present study

#### Purpose

The general aim of the present study is to enrich the literature on people’s willingness to use telemedicine services and to provide policy decision makers valuable information about the sub-Saharan African population’s willingness to use tele-consultation public health services. Specifically, this study aims at accessing the Mozambican people’s willingness to use tele-consultation public health services and the determinant factors associated with their willingness.

#### Country characteristics

Located in Southern Africa, Mozambique has a population of 28.8 million [31]. In 2020, the country Human Development Index was 0.456, ranking it among the 10 poorest nations in the world [32]. The coverage rate of basic public health services and the ratio of physician per 1000 inhabitants are among the lowest in the world (22 and 0.1%, respectively) [1, 2]. In 2018, the Mozambican ministry of health, in partnership with the ministry of science and technology launched a tele-health centre (Telehealth MZ) [33]. Initially, its purpose is to assist public health professionals in the provision of healthcare services to the population, giving priority to primary healthcare, which includes preventive and community actions. In the near future, the tele-health centre will be used to develop tele-consultation projects [33].

As the Mozambican government is planning to develop in the near future tele-consultation projects to increase the population’s accessibility to the public health service, this study is envisaged to be able to give the policy decision-makers useful knowledge.

#### Concept definition

The participant’s *willingness* to use teleconsultation was conceived as the desire to use and agreement to use, and its antonym of the unwillingness to use or the reluctance to use. The *willingness determinant factors* refer to contextualised situations described in the Materials and Design sub-section.

#### Research questions

The main question was:

1. To what extent are Mozambican adults willing to use tele-consultation in public health services? Could that willingness be translated in terms of different perspectives (clusters) according to the health contextualised situations that the participants have to face?

The subsidiary questions were:

2. Are there significant differences in levels of willingness to use tele-consultation between the participant’s different groups and across the different health problem situations (e.g., female vs. male participants, urban vs. rural participants, religious practitioners vs. non-religious practitioners; in case of first consultation vs. follow-up consultation, the same price as face-to-face consultation vs. cheaper than in face-to-face consultation, mild health problem vs. severe health problem, organic/infectious health problem vs. psychological health problem)?
3. What will be the effects of sociodemographic characteristics on the Mozambican participant’s willingness different perspectives regarding the use of tele-consultation public health services?
4. What are the contextualised health situations effects on the participants’ different perspectives regarding their willingness to use tele-consultation in public health services?

The underlying hypothesis at the base of this study design was that several perspectives (clusters) on the willingness to use tele-consultation services exist among the Mozambican adult population, and that these different perspectives are related to the sociodemographic characteristics such as age, sex, socioeconomic status, education level, living area, religion, and the level of perceived stress. Indeed, previous studies investigating the willingness of Mozambican people’s willingness to take HIV prophylaxis drugs and to get vaccinate against malaria have shown different positions related to the same sociodemographic characteristics [34, 35].

## Methods

### Participants

Participants were unpaid volunteers recruited by students working as research assistants.

First, in the provinces of Maputo, Gaza and Inhambane, we selected, among all city districts (in urban areas) and among all villages (in rural areas), those whose population presented more sociodemographic diversity. Second, students who knew these districts well were trained as research assistants. These assistants went door to door in the chosen cities, towns, and villages, looking for individuals over 17 years-old who would be willing to participate in the study. Of the 578 contacted individuals who qualified, 403 agreed to participate (204 females and 199 males). Following the signing of an informed consent, an appointment for the participant to complete the data collection material was set up. The data were collected from May 2019 to March 2020. The choice of this recruitment method was made to rationalise the limited financial means we had to conduct this research while trying to have a sample that is as representative as possible of the adult population of the country. Table 1 displays the participants’ demographic characteristics.

**Table 1:** The associations between the five clusters and the socio-demographic characteristics

Characteristics	Clusters					Total
	never-will *n* = 59	severity *n* = 105	consul-cat *n* = 90	undecided *n* = 65	price-severity *n* = 84	
Age, years						
youth (18–26)	4 (6.8)^a^	45 (42.9)^a^	36 (40)^a^	27 (41.5)^a^	29 (34.5)^a^	141
adults (27–35)	12 (20.3)^a^	35 (33.3)^a^	22 (24.4)^ab^	14 (21.5)^ab^	19 (22.6)^a^	102
mature (36–66)	43 (72.9)^a^	25 (23.8)^a^	32 (35.6)^b^	24 (36.9)^b^	36 (42.9)^a^	160
Sex						
male	8 (13.6)^a^	83 (79)^a^	40 (44.4)^a^	30 (46.2)^a^	38 (45.2)^a^	199
female	51 (86.4)^a^	22 (21)^a^	50 (55.6)^a^	35 (53.8)^b^	46 (54.8)^b^	204
Education, years						
primary (< 12)	17 (28.8)^a^	6 (5.7)^a^	5 (5.6)^a^	31 (47.7)^a^	18 (21.4)^a^	77
secondary (12–14)	31 (52.5)^a^	44 (41.9)^a^	51 (56.7)^a^	26 (40)^a^	60 (71.4)^a^	212
tertiary (> 14)	11 (18.6)^a^	55 (52.4)^a^	34 (37.8)^a^	8 (12.3)^a^	6 (7.1)^a^	114
Socioeconomic level						
low	40 (67.8)^a^	33 (31.4)^a^	48 (53.3)^a^	54 (83.1)^a^	74 (88.1)^ab^	249
intermediate	15 (25.4)^a^	51 (48.6)^a^	33 (36.7)^a^	10 (15.4)^a^	7 (8.3)^a^	116
high	4 (6.8) ^a^	21 (20)^a^	9 (10)^a^	1 (1.5)^a^	3 (3.6)^b^	38
Perceived stress						
low	51 (86.4)^a^	6 (5.7)^a^	12 (13.3)^a^	26 (40)^a^	17 (20.2)^a^	112
intermediate	0 (0)	53 (50.5)^a^	24 (26.7)^a^	8 (12.3)^a^	19 (22.6)^b^	104
high	8 (13.6)^a^	46 (43.8)^a^	54 (60)^a^	31 (47.7)^a^	48 (57.1)^ab^	187
Religion						
christian	26 (44.1)^a^	59 (56.2)^a^	54 (60)^a^	19 (29.2)^a^	34 (40.5)^a^	192
muslim and others	12 (20.3)^ab^	38 (36.2)^a^	26 (28.9)^a^	6 (9.2)^a^	13 (15.5)^abc^	95
traditional-african	21 (35.6)^b^	8 (7.6)^a^	10 (11.1)^a^	40 (61.5)^a^	37 (44)^b^	116
Practitioner believers						
practitioners	28 (47.5)^a^	68 (64.8)^a^	53 (58.9)^a^	10 (15.4)^a^	20 (23.8)^a^	179
non-practitioners	31 (52.5)^a^	37 (35.2)^a^	37 (41.1)^a^	55 (84.6)^a^	64 (76.2)^a^	224
Living area						
urban	37 (62.7)^a^	93 (88.6)^a^	70 (77.8)^a^	26 (40)^a^	48 (57.1)^a^	274
rural	22 (37.3)^a^	12 (11.4)^a^	20 (22.2)^a^	39 (60)^a^	36 (42.9)^a^	129

*Never-will* never willing, *consul-cat* consultation category

Data are shown as *n* (%). Figures with the same exponent in each column are significantly different (*p* < 0.05). For example: regarding perceived stress, 20.2% is significantly different from 57.1, 22.6% is significantly different from 57.1, and 20.2% and 22.6% are not significantly different; they have different exponents

### Material and design

First, it must be stressed that the study was based on the premises that a tele-consultation programme was set up and shall be operated by the national health service alongside face-to-face health services. The purpose was to assess the participants’ willingness to use that tele-consultation service as a function of a set of contextualised health situations (determinants factors).

The research material was written and presented to the participants in Portuguese, which is the main official language in Mozambique (see the English language version of the study data collection material in the additional file 1). It consisted of 32 cards containing a vignette (scenarios) of a few lines, a question, and a response scale. Five factors corresponding to five contextualised health situations were systematically combined in these vignettes, including: *the consultation category* (first consultation vs. follow-up consultation); *the health problem category* (infectious/organic vs. psychological); *the health problem severity* (mild / easily curable disease vs. severe / not easily controlled disease); *the physician category* (general practitioner vs. specialist); *the consultation price* (25% cheaper than face-to-face consultation vs. as expensive as face-to-face consultation). Thus, these 32 conditions constituted a 2 × 2 × 2 × 2 × 2 within-subjects design. These vignettes were specially developed for this study according to the procedure recommended by Anderson [36, 38].

The following is a vignette example: “*Maria has not been feeling well for the last few days. The symptoms (fever, cold, cough, and diarrhea) indicate that she may have contracted an easily curable seasonal illness such as the flu or gastroenteritis. With the goal of making an appointment to have a consultation with a health professional as quickly as possible, Marie contacted (around 8 a.m.) the public health services closest to her home. Due to the lack of availability of health personnel working on-site and the overcrowding at the health care centre, she was offered either an on-site consultation with a generalist physician but scheduled for 3 p.m. or a remote consultation (tele-consultation) scheduled for 10 a.m. (in a mobile truck parked in her neighborhood and equipped with video conferencing and medical diagnostic tools). Following this remote consultation, if necessary, the generalist physician will immediately send her an electronic medical prescription by telephone so that she can go to the pharmacy to get medications or a prescription to go for additional examinations at the nearest medical examination centre. The price of the remote consultation would be the same as the price of the face-to-face consultation. If you were Maria to what extent would you be willing to use the tele-consultation service proposed to her?*”. The difference between face-to-face and the tele-consultation is that in the latter case Maria will not pay for transport to the centre and she will be able to consult the doctor 5 h earlier. Participants’ willingness to use the proposed tele-consultation health service under each of the 32 conditions was assessed using an 11-point response scale, ranging from 0 (“certainly would not”) to 10 (“certainly would”).

The study vignettes were developed as follow. First, 20 Mozambican physicians (10 generalist and 10 specialist) were asked to list contextualised situations (determinant factors) associated with the search of public health services in this country. The synthetisation of the list resulted in 12 situations. Second, a pilot research was conducted in which 67 participants (30 women, 37 men, none of whom participated in the main study) were asked to evaluate the 12 factors/situations for their relevance and potential impact on Mozambican tele-consultation decision making. As a result of the pilot study, five factors were chosen as relevant and used to develop the vignettes.

In addition to the 32 vignettes, the participants completed the Cohen & Williamson perceived stress scale [37]. The basis for adding the Cohen & Williamson perceived stress scale stems from the fact that some previous studies related to public health issues conducted in Mozambique in recent years have indicated that the decision making concerning the willingness to consult a physician, to take certain medications, get tested for a disease is associated with the level of perceived stress [34, 35].

### Procedure

The ethics committee of the first author affiliated university approved the study. All participants gave their informed consent, and their full anonymity has been guaranteed. The procedure was the one suggested by Anderson [36, 38]. The participants’ data collection was a quiet room at their homes. Processing was strictly individual. Participants were given a brief synopsis of the research methods and of the purpose of the study. They were also given instructions on how to use the response scale. They could ask questions and the researchers answered them in a way that was not suggestive of any particular response. Vignettes were randomly ordered for each participant. It usually took between 55 and 75 min to rate the 32 vignettes. Participants answered additional questions about their sex, age, socioeconomic status (SES, determined by the family monthly income), educational level (determined by years of schooling), religion and completed the perceived stress scale.

### Statistical analyses

All statistical analyses were conducted using R.

To answer the first research question, a cluster analysis (k-means method) was performed on the whole set of raw data. The optimal number of clusters was determined using the *NbClust* R package, which runs several tests and draws a conclusion according to the majority rules.

To answer the second research question, a Welch two-sample *t*-test was run to compare the participant’s different groups and the different health problem situations mean ratings (e.g., female participants’ mean ratings vs. male mean ratings; participants’ from rural areas mean ratings vs. participants’ from urban area mean ratings, etc.).

To answer the third research question, Pearson’s chi-squared test was conducted to test the effects of demographic characteristics on the participants’ different perspectives regarding the use of tele-consultation public health services.

To answer the fourth research question, separate ANOVAs were conducted on the data of each cluster, using a *consultation category* x *health problem category* x *health problem severity* x *physician category* x *consultation price*, 2 × 2 × 2 × 2 × 2 design. Due to the great number of comparisons, the significance threshold was set at 0.001.

## Results

**Fig. 1:**
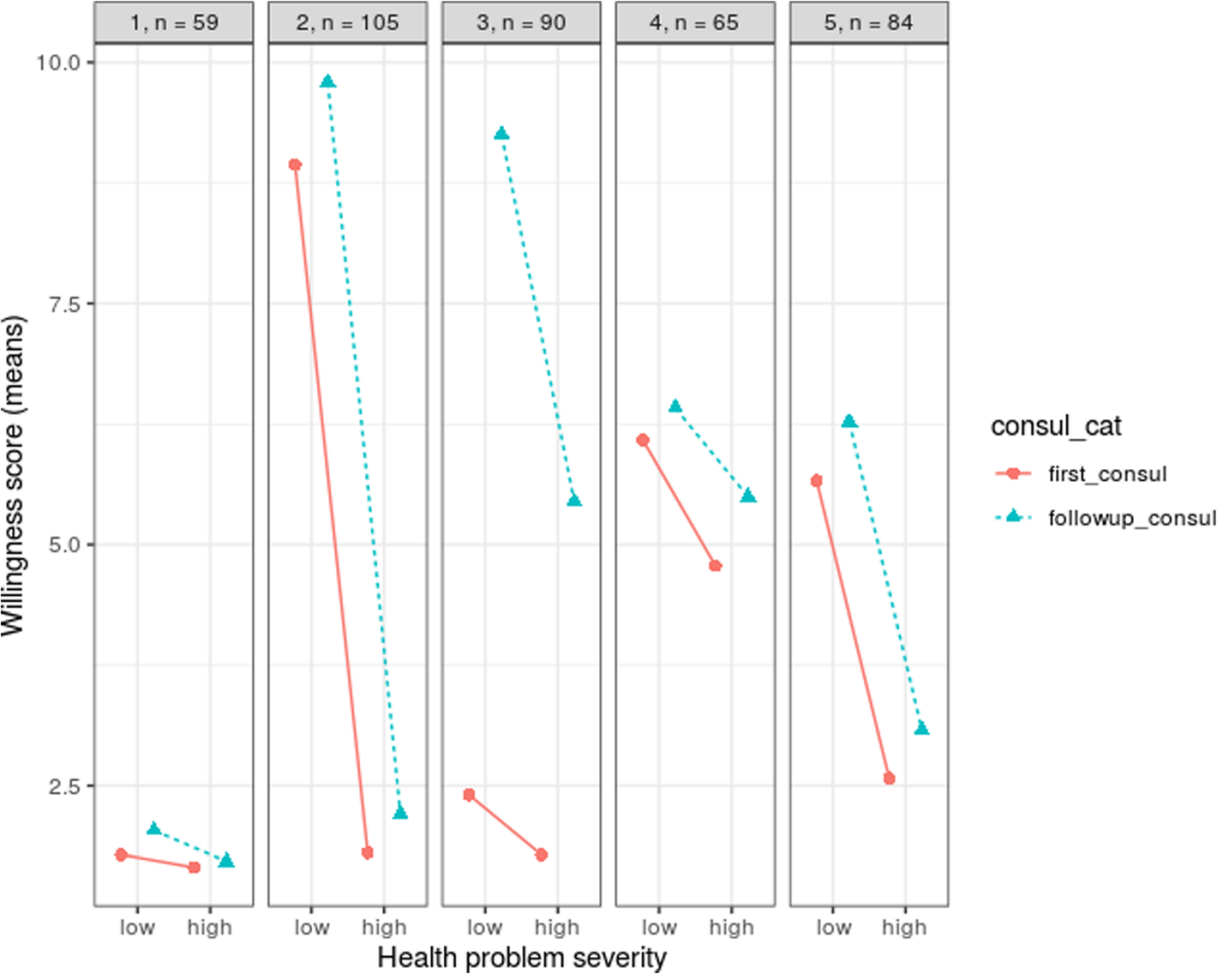
The visualization of the data pattern corresponding to the five clusters. In each panel, willingness to use tele-consultation public health services is on the vertical axis, and the two levels of the health problem severity (hp_severity) factor are on the horizontal axis. The two curves correspond to the two levels of consultation category (consul_cat). Each panel corresponds to one perspective (cluster): 1 = never-willing (15%), 2 = severity (26%), 3 = consultation-category (22%), 4 = undecided (16%), 5 = price-severity (21%)

**Fig. 2:**
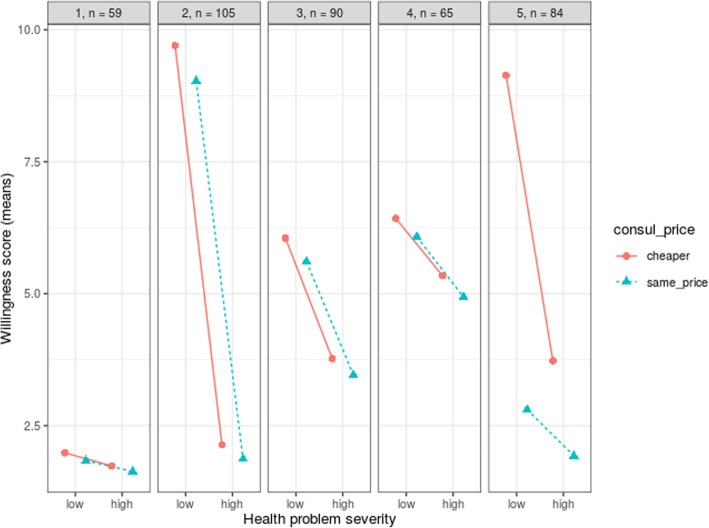
The visualization of the data pattern corresponding to the five clusters. In each panel, willingness to use tele-consultation public health services is on the vertical axis, and the two levels of the health problem severity (hp_severity) factor are on the horizontal axis. The two curves correspond to the two levels of consultation price (consul_price). Each panel corresponds to one perspective (cluster): 1 = never-willing (15%), 2 = severity (26%), 3 = consultation-category (22%), 4 = undecided (16%), 5 = price-severity (21%)

Table 1 shows the number and percentage of participants in each cluster and in each condition for the various categorisations. Table 2 displays the main results of the ANOVA conducted between the five clusters and the modeled factors. Figures 1 and 2 exhibit the main patterns of data corresponding to the five clusters.

**Table 2:** The results of the ANOVA conducted between clusters and the modeled factors

	*df*	*MS*	*F*	*p*	η^2^_p_
Cluster never-willing					
consul_cat	1	11.44	10.13	.001	.01
consul_price	1	7.50	6.64	.010	.05
physician_cat	1	.11	.10	.745	.00
hp_severity	1	24.03	21.28	.000	.17
hp_cat	1	1.17	1.03	.300	.01
Cluster severity					
consul_cat	1	328	193.30	.000	.01
consul_price	1	183	107.77	.000	.05
physician_cat	1	7	4.05	.044	.05
hp_severity	1	45,511	26,812.06	.000	.88
hp_cat	1	164	96.53	.000	.05
consul_cat x consul_price	1	13	7.58	.005	.22
consul_cat x hp_severity	1	43	25.05	.000	.05
consul_price x hp_severity	1	34	19.79	.000	.05
hp_severity x hp_cat	1	16	9.60	.001	.01
Cluster consultation-category					
consul_cat	1	19,897.5	6951.17	.000	.59
consul_price	1	102	35.6341	.000	.01
physician_cat	1	8	2.80	.094	.01
hp_severity	1	3533.4	1234.37	.000	.30
hp_cat	1	19.3	6.75	.009	.01
consul_cat x hp_severity	1	1827.2	638.34	.000	.18
Cluster undecided					
consul_cat	1	141.75	37.96	.000	.02
consul_price	1	73.50	19.68	.000	.02
physician_cat	1	.06	.01	.900	.01
hp_severity	1	645.81	172.9737	.000	.08
hp_cat	1	85.21	22.82	.000	.06
consul_cat x hp_severity	1	19.42	5.20	.002	.01
hp_severity x hp_cat	1	36.89	9.88	.001	.01
Cluster price-severity					
consul_cat	1	208.1	77.64	.000	.03
consul_price	1	11,115	4145.94	.000	.61
physician_cat	1	3.4	1.27	.258	.01
hp_severity	1	6637.7	2475.89	.000	.48
hp_cat	1	57.2	21.32	.000	.01
consul_price x hp_severity	1	2	.75	.000	.33
hp_severity x hp_cat	1	14	5.22	.002	.01

Only the significant interactions are reported in this table

*df* degree of freedom, *η*^*2*^_*p*_ effect size, *consul_cat* consultation category, *consul_priece* consultation price, *physician_cat* physician category, *hp_severity* health problem severity, *hp_cat* health problem category

### Mean (M) comparison results

Male participants indicated a significatively higher willingness to use proposed tele-consultation health services than their female counterparts (*M* = 5.18 vs. 4.10; *t* = 7.68; *p* = 0.000). Young participants (18–35 years old) showed higher willingness than older participants (> 35 years old) (*M* = 5.07 vs. 4.16; *t* = 5.39; *p* = 0.000). Participants with low SES indicated lower willingness than participants with high SES (*M* = 5.07 vs. 4.16; *t* = − 2.52; *p* = 0.015). Participants with low education level showed lower willingness than participants with high education level (*M* = 4.45 vs. 5.17; *t* = − 3.23; *p* = 0.001). Participants with low perceived stress showed higher willingness than participants with higher perceived stress (*M* = 5.37 vs. 3.41; *t* = 10.25; *p* = 0.000). There was no mean significant difference of willingness between religious non-practitioners and religious practitioners or between the participants from different religions. In addition, there was no mean significant difference in willingness ratings between the participants from urban and those from rural areas.

The first tele-consultation condition resulted in significant lower willingness ratings than the follow-up tele-consultation condition (*M* = 3.86 vs. 5.42; *t* = − 11.84; *p* = 0.000). The same price as face-to-face consultation condition resulted in lower willingness compared with the condition in which the tele-consultation was 25% cheaper than face-to-face consultation (*M* = 4.07 vs. 5.20; *t* = − 9.23; *p* = 0.000). The mild health problem condition resulted in higher willingness ratings than the severe health problem condition (*M* = 6.28 vs. 2.99; *t* = 22.16; *p* = 0.000). The organic/infectious health problem condition led to higher willingness mean ratings than the psychological health problem condition (*M* = 6.28 vs. 2.99; *t* = 2.61; *p* = 0.009). There was no significant difference in willingness mean ratings between the generalist physician and the specialist physician condition.

### Cluster and chi-squared analysis results

The optimal number of clusters was set to five.

The first cluster (15% of the sample) was called *never-willing*. On the 11-point scale, almost all ratings were close to 0–1 points (*M* = 0.74). However, the ratings were slightly higher when the health problem was mild (*M* = 1.57) than when it was severe (*M* = 0.56). As it can be seen in Table 1, older, female, Christian, urban, and highly stressed participants were more often present in this cluster, compared with male, younger, Muslim, rural, lowly stressed participants.

The second cluster (26% of the sample) was called *severity*, because the health problem severity factor explained most of the variance. Ratings were higher when the health problem was mild (*M* = 8.36) than when it was severe (*M* = 1.78). More educated, young, wealthier, religious practitioners, Christian, Muslim, and participants from urban areas were more often members of this cluster, compared with older, less educated, poorer, religious non-practitioners, practitioners of traditional African religions participants, and those from rural areas.

The third cluster (22% of the sample) was called *consultation-category*, because the consultation category factor explained most of the variance: namely, ratings for willingness were considerably higher in the follow-up consultation situation (*M* = 7.49) than in the first consultation cases (*M* = 1.68). Besides, ratings for willingness were considerably higher when: (1) the health problem was mild (*M* = 8.94) than when the health problem was severe (*M* = 5.77); (2) the willingness was rated higher when the tele-consultation was cheaper than the face-to-face consultation (*M* = 5.29). The willingness was lower when the tele-consultation costs were the same as the face-to-face consultation (*M* = 4.23). Better educated, urban, more stressed, religious practitioners’ participants were more often members of this cluster, compared with less educated, rural, less stressed participants, and religious non-practitioners; Christians and Muslim were more often members of this cluster, compared with non-Christians and non-Muslim participants.

The fourth cluster (16% of the sample) was called *undecided*, because the configuration of factors leading to the participants willingness to use the tele-consultation could not be determined (nearly all of their willingness ratings were close to the middle of the scale) (*M* = 4.46), although these ratings were slightly higher when the health problem was mild (*M* = 5.07) than when they were severe (*M* = 3.88). As it can be seen in Table 1, less educated participants, traditional African religions believers and participants who reported low SES were more often members of this cluster, compared with better educated, Christians and Muslim, and participants who reported a high SES. Participants from rural areas and religious non-practitioners were also more often members of this cluster compared with urban and religious practitioners’ participants.

The fifth cluster (21% of the sample) was called *price-severity* because two factors (consultation price and health problem severity) explained most of the variance and they interacted strongly. Ratings were higher when the tele-consultation price was cheaper compared with the price of a face-to-face consultation (*M* = 5.20) than when it was the same (*M* = 4.07), and when the health problem was mild (*M* = 7.84) than when it was severe (*M* = 2.94). In addition, ratings were slightly higher in the case of follow-up consultation (*M* = 5.37) and in the case of psychological health problem (*M* = 5.28) than in the case of first consultation (*M* = 4.93) and organic/infectious health problem (*M* = 5.07). In the best-case scenario, i.e., cheaper price and mild health problem, the participants’ mean rating was 9.13. Less educated and poorer participants, religious non-practitioners and participants who reported a high level of perceived stress were more often members of this cluster, compared with better educated and wealthier participants, religious practitioners, and participants who reported a low level of perceived stress.

### ANOVA results

As it can be seen in Table 2, among the five factors used in the vignettes as hypothetical health situations (health problem severity, consultation category, consultation price, health problem category and physician category), the first had a significant and strong effect on all five clusters; the latter had no significant effect on any of the five factors; the second and the third had significant effects (small and moderate) on all five clusters; the fourth factor had significant effect (small) on all five cluster except the *never-willing* cluster.

## Discussion

### Main results and interpretation

First, the results of this study showed that, in general, female, less educated, poorer, and participants reporting high levels of perceived stress were relatively less willing to use tele-consultation public health services compared with male, more educated and wealthier participants. One possible explanation of this difference may lie in the fact that, in the Mozambican context, as it is the case in other parts of the world, female individuals are generally more subjected to complicated health problem requiring special physical examination (e.g., gynaecologic health issues, sexually transmitted infections, psychological and physical trauma and stress due to the pervasiveness of the violence against women), compared with their male counterparts [2]. For this reason, women might feel the need for face-to-face consultation more than men, because women may see this type of consultation as more suitable for the special attention provided by the physician in terms of examination, privacy issues, reassurance, support and guidance. Generally, Mozambican women are also less educated, have less economic resources and are more subject to stress comparatively to Mozambican men [39]. Less educated, poorer, and highly stressed individuals are often those who are more subjected to complicated health problem that may require a special attention as mentioned above; and they also may have some concerns linked to the unfamiliarity with the technology used in tele-consultation, absolute need for physical examination and face-to-face interaction. Indeed, in previous studies [30], these last two elements have been reported as barriers to the acceptability of tele-consultation health services.

Second, this study findings indicate that several (five) qualitatively different perspectives regarding willingness to use tele-consultation public health services exist among the participants. These positions are related to the participants’ sociodemographic characteristics. Particularly, according to the study results:

(a) A relatively small number of participants (15%) indicated that they would be unwilling to use the proposed tele-consultation public health services irrespective of the contextualised health situations, the *never-willing* perspective. In light of the method used in this study, this means that, when given a chose, these participants would highly prefer face-to-face consultation than tele-consultation. The fact that participants who endorse this position are more often female than male, poorer than wealthier, less educated than more educated may be explain by the same reasons laid out in the first paragraph of this discussion.

(b) A large majority of the participants (69% = the *severity*, *consultation-category* and *price-severity* clusters) suggested that they would be highly willing to use the proposed tele-consultation public health services provided that the health issue they would suffer from is mild, in case of follow-up medical consultation, and in situation where the tele-consultation is cheaper than the face-to-face consultation. The participants embracing this position are often younger, more educated, wealthier, urban, and report lower levels of perceived stress than their counterparts. In particular, the participant’s age, level of education and perceived stress are great predictors of the willingness level to use tele-consultation health services. The category of participants reporting higher willingness are relatively more likely to be familiar with the technology used in tele-consultation such as smartphones, videoconference tools or remote interaction web application; therefore, these participants may have less apprehension related to the use of the technology associated with tele-consultations. Also, given their education and SES level, these participants may have resources allowing them to better manage the situations associated with the onset and development of health problems; thus, they may have less need for the kind of attention, reassurance, guidance that only a face-to-face interaction can provide. Similar findings have been reported in studies carried out in other regions of the globe (see Introduction section).

(c) Finally, a relatively small group of participants (16%) were undecided. Clusters reporting participants’ indecisiveness have been found in some previous studies. According to Johnston Conover et al. [40], this indeterminability may be related to perceived lack of information/competence, genuine uncertainty, difficulties in publicly asserting preferences, and reaction against what is perceived as an intrusion into someone’s privacy. Considering the methodology used in this study, this particular finding can also be interpreted as an ambivalence regarding the use of the proposed tele-consultation services. In contrast, if the participants in this cluster had been asked to give response to a single question or to a single vignette, their responses would be interpreted as a medium level of willingness to use the proposed tele-consultation services.

### Strengths and limitations

Among the current study strengths, we can mention the data collection method based on scenarios describing realistic, multifactorial, and intra-subject contextualised situations. Indeed, this method made it possible to identify and combined health situations in which the participants would be willing or not to use the tele-consultation public health services. In addition, multifactorial scenarios made it possible to standardize the situations between the participants, to examine how people combine and weight the different factors to take a position on the issue under study. Among the present study limitations, we can mention the use of a convenience sample. Although we have made sure that all ethnic, socio-cultural, and socio-economic categories are represented, we did not use pure representative sampling techniques. Also, because the vignette technique is time consuming, despite our recruitment efforts, we were unable to recruit more than 403 participants. These two elements combined raise some uncertainty regarding the generalization of the results to the whole Mozambican population.

### Open questions and indications for future research

To reach the UN goal of universal health coverage by 2030, different tele-consultation pilot projects are underway across sub-Saharan Africa [10]. To inform policymakers and enrich the telemedicine literature, this research was designed to examine Mozambican people willingness to use tele-consultation and the determinants associated to their willingness. However, the situations (determinants) included in the study are not exhaustive. For instance, would factors such as the distance an individual usually has to travel from home to the closest face-to-face consultation be predictive of the level of his willingness to use tele-consultation public services in order to save time and money? More studies must be conducted, including much more factors and situations likely to predict the participants’ level of willingness to use public tele-consultation health services. Future studies must also include larger participant samples and diversify the methodology to cover broader aspects of the concerned populations social and cultural life.

### Conclusion and recommendations for policymakers

The main results of this study suggest that the majority of participants (69%) are highly willing to use tele-consultation public health services in the case of mild illness, cheaper prices and follow-up consultation. However, it seems that individuals that are more in need of public health services (individuals from rural areas, women, less educated and poor individuals) indicated a preference for face-to-face consultation. This means that before rolling out permanent and large scale public tele-consultation health service, policymakers must primarily remove social, cultural and technological barriers likely to inhibit the use of these services by the category of population for which these services are directed in the first place. In addition, it would be advisable to start by proposing tele-consultation public health services for mild health issues and follow-up consultation, while making these remote services cheaper than the face-to-face ones. After the publication of this article, a document indicating the conclusion of the study and stressing the need to conduct a new study on a larger scale will be sent to the various bodies responsible for advising the Mozambican authorities in the implementation of public health policies.

## Supplementary Information

**Additional file 1.** The material used for the study data collection. This additional file contains the English language version of (1) the 32 vignettes used as the main material for data collection, (2) the Cohen & Williamson perceived stress scale [38] used to measure the participants level of perceived stress, and (3) the sociodemographic items used to gather the participants’ additional information.

## Data Availability

The material used in this study (in Portuguese and in French) and the data supporting this finding are available from the corresponding author upon request. The dataset analysed during the current study is also available in the Open Science Framework repository, weblink: https://osf.io/3gfsr/quickfiles

## Abbreviations

UHCI: Universal Health Coverage Index
HIV: Human Immunodeficiency Virus
AIDS: Acquired Immunodeficiency Syndrome
WHO: World Health Organization
COVID-19: Coronavirus Disease 2019
SES: socioeconomic status
M: mean
ANOVA: Analysis of Variance
DF: Degree of Freedom
MS: Mean Squared

## Footnotes

1 Going, respectively, from 32% in 2014 to 59% in 2018 and from 13% in 2014 to 24% in 2018.
