# Supplementary material for: People’s willingness and determinants to use selected tele-consultation public health services in Mozambique
Source: BMC Public Health. 2021 May 19;21:947. doi: 10.1186/s12889-021-10709-9 (PMC8132030; doi:10.1186/s12889-021-10709-9)
Supplement: Supplementary file 1 — Additional file 1. The material used for the study data collection. This additional file contains the English language version of (1) the 32 vignettes used as the main material for data collection, (2) the Cohen & Williamson perceived stress scale [38] used to measure the participants level of perceived stress, and (3) the sociodemographic items used to gather the participants’ additional information. [file 12889_2021_10709_MOESM1_ESM.docx]

**The material used for the study data collection^[[1]](#footnote-1)^**

1. **THE 32 VIGNETTES**

**Study design**

Factorial design: 2 x 2 x 2 x 2 x 2 = 32 vignettes (scenarios).

Factors: consultation_category (first vs follow_up); consultation_price (same price vs 25% cheaper); physician_category (generalist physician vs specialist physician); health_problem_severity (severe vs mild); health_problem_category (organic/infectious vs psychological).

**Procedure**

The study purpose is explained to the participants. The study material completion instructions are laid out. The participants are encouraged to ask questions for clarifications. Then, the vignettes are presented to the participants one by one in random order. The participants are required to read the vignettes and rate their willingness using the 11-point scale at the end of each vignette.

**Factor 1 + factor 5 variation**

**Vignette 1**

Maria has not been feeling well for the last few days. The symptoms (fever, cold, cough, and diarrhea) indicate that she may have contracted an easily curable seasonal illness such as the flu or gastroenteritis. With the goal of making an appointment to have a consultation with a health professional as quickly as possible, Marie contacted (around 8 a.m.) the public health services closest to her home. Due to the lack of availability of health personnel working on site and the overcrowding at the health care center, she was offered either an on-site consultation with a *generalist physician* but scheduled for 3 p.m. or a remote consultation (tele-consultation) scheduled for 10 a.m. (in a mobile truck parked in her neighborhood and equipped with video conferencing and medical diagnostic tools). Following this remote consultation, if necessary, the *generalist physician* will immediately send her an electronic medical prescription by telephone so that she can go to the pharmacy to get medications or a prescription to go for additional examinations at the nearest medical examination center. The price of the remote consultation would be the same as the price of the face-to-face consultation.

*If you were Maria to what extend would you be willing to use the tele-consultation service proposed to her*?

Certainly would not o-----o-----o-----o-----o-----o-----o-----o-----o-----o-----o Certainly would

**Vignette 2**

Since Bernard learned two weeks ago that he had been selected for a job interview he has not been feeling well. The symptoms (slight increase in blood pressure, difficulty falling asleep, involuntary muscle contraction) indicate that he is experiencing an anxiety disorder. With the goal of making an appointment to have a consultation with a health professional as quickly as possible, Bernard contacted (around 8 a.m.) the public health services closest to his home. Due to the lack of availability of health personnel working on site and the overcrowding at the health care center, he was offered either an on-site consultation with a *generalist physician* but scheduled for 3 p.m. or a remote consultation (tele-consultation) scheduled for 10 a.m. (in a mobile truck parked in his neighborhood and equipped with video conferencing and medical diagnostic tools). Following this remote consultation, if necessary, the *generalist physician* will immediately send him an electronic medical prescription by telephone so that she can go to the pharmacy to get medications or a prescription to go for additional examinations at the nearest medical examination center. The price of the remote consultation would be the same as the price of the face-to-face consultation.

*If you were Bernard to what extend would you be willing to use the tele-consultation service proposed to him*?

Certainly would not o-----o-----o-----o-----o-----o-----o-----o-----o-----o-----o Certainly would

**---------------------------------------------------------------------------------------------------------------------------**

**Vignette 3**

For two weeks Manuel has not been feeling well. The symptoms (a loss of about 10kg in weight, gastroenteritis, skin lesions visible all over the body) indicate that he may have a serious disease (organic/infectious). With the goal of making an appointment to have a consultation with a health professional as quickly as possible, Manuel contacted (around 8 a.m.) the public health services closest to his home. Due to the lack of availability of health personnel working on site and the overcrowding at the health care center, he was offered either an on-site consultation with a *generalist physician* but scheduled for 3 p.m. or a remote consultation (tele-consultation) scheduled for 10 a.m. (in a mobile truck parked in his neighborhood and equipped with video conferencing and medical diagnostic tools). Following this remote consultation, if necessary, the *generalist physician* will immediately send him an electronic medical prescription by telephone so that she can go to the pharmacy to get medications or a prescription to go for additional examinations at the nearest medical examination center. The price of the remote consultation would be the same as the price of the face-to-face consultation.

*If you were Manuel to what extend would you be willing to use the tele-consultation service proposed to him*?

Certainly would not o-----o-----o-----o-----o-----o-----o-----o-----o-----o-----o Certainly would

**Vignette 4**

Since the death of her husband, two weeks ago, in a car accident, Sofia is not feeling well. The symptoms (severe depression, multiple panic attacks, suicide attempt) indicate that she may be experiencing a very serious mental health problem. With the goal of making an appointment to have a consultation with a health professional as quickly as possible, Sofia contacted (around 8 a.m.) the public health services closest to her home. Due to the lack of availability of health personnel working on site and the overcrowding at the health care center, she was offered either an on-site consultation with a *generalist physician* but scheduled for 3 p.m. or a remote consultation (tele-consultation) scheduled for 10 a.m. (in a mobile truck parked in her neighborhood and equipped with video conferencing and medical diagnostic tools). Following this remote consultation, if necessary, the *generalist physician* will immediately send her an electronic medical prescription by telephone so that she can go to the pharmacy to get medications or a prescription to go for additional examinations at the nearest medical examination center. The price of the remote consultation would be the same as the price of the face-to-face consultation.

*If you were Sofia to what extend would you be willing to use the tele-consultation service proposed to her*?

Certainly would not o-----o-----o-----o-----o-----o-----o-----o-----o-----o-----o Certainly would

**Factor 1 + factor 5 variation and factor 2 second modality**

**Vignette 5**

Bernadette has not been feeling well for the last few days. The symptoms (fever, cold, cough, and diarrhea) indicate that she may have contracted an easily curable seasonal illness such as the flu or gastroenteritis. With the goal of making an appointment to have a consultation with a health professional as quickly as possible, Bernadette contacted (around 8 a.m.) the public health services closest to her home. Due to the lack of availability of health personnel working on site and the overcrowding at the health care center, she was offered either an on-site consultation with a *specialist physician* but scheduled for 3 p.m. or a remote consultation (tele-consultation) scheduled for 10 a.m. (in a mobile truck parked in her neighborhood and equipped with video conferencing and medical diagnostic tools). Following this remote consultation, if necessary, the *specialist physician* will immediately send her an electronic medical prescription by telephone so that she can go to the pharmacy to get medications or a prescription to go for additional examinations at the nearest medical examination center. The price of the remote consultation would be the same as the price of the face-to-face consultation.

*If you were Bernadette to what extend would you be willing to use the tele-consultation service proposed to her*?

Certainly would not o-----o-----o-----o-----o-----o-----o-----o-----o-----o-----o Certainly would

**Vignette 6**

Since Antonio learned two weeks ago that he had been selected for a job interview he has not been feeling well. The symptoms (slight increase in blood pressure, difficulty falling asleep, involuntary muscle contraction) indicate that he is experiencing an anxiety disorder. With the goal of making an appointment to have a consultation with a health professional as quickly as possible, Antonio contacted (around 8 a.m.) the public health services closest to his home. Due to the lack of availability of health personnel working on site and the overcrowding at the health care center, he was offered either an on-site consultation with a *specialist t physician* but scheduled for 3 p.m. or a remote consultation (tele-consultation) scheduled for 10 a.m. (in a mobile truck parked in his neighborhood and equipped with video conferencing and medical diagnostic tools). Following this remote consultation, if necessary, the *specialist physician* will immediately send him an electronic medical prescription by telephone so that she can go to the pharmacy to get medications or a prescription to go for additional examinations at the nearest medical examination center. The price of the remote consultation would be the same as the price of the face-to-face consultation.

*If you were Antonio to what extend would you be willing to use the tele-consultation service proposed to him*?

Certainly would not o-----o-----o-----o-----o-----o-----o-----o-----o-----o-----o Certainly would

**---------------------------------------------------------------------------------------------------------------------------**

**Vignette 7**

For two weeks João has not been feeling well. The symptoms (a loss of about 10kg in weight, gastroenteritis, skin lesions visible all over the body) indicate that he may have a serious disease (organic/infectious). With the goal of making an appointment to have a consultation with a health professional as quickly as possible, João contacted (around 8 a.m.) the public health services closest to his home. Due to the lack of availability of health personnel working on site and the overcrowding at the health care center, he was offered either an on-site consultation with a *specialist physician* but scheduled for 3 p.m. or a remote consultation (tele-consultation) scheduled for 10 a.m. (in a mobile truck parked in his neighborhood and equipped with video conferencing and medical diagnostic tools). Following this remote consultation, if necessary, the *specialist physician* will immediately send him an electronic medical prescription by telephone so that she can go to the pharmacy to get medications or a prescription to go for additional examinations at the nearest medical examination center. The price of the remote consultation would be the same as the price of the face-to-face consultation.

*If you were João to what extend would you be willing to use the tele-consultation service proposed to him*?

Certainly would not o-----o-----o-----o-----o-----o-----o-----o-----o-----o-----o Certainly would

**Vignette 8**

Since the death of her husband, two weeks ago, in a car accident, Tánia is not feeling well. The symptoms (severe depression, multiple panic attacks, suicide attempt) indicate that she may be experiencing a very serious mental health problem. With the goal of making an appointment to have a consultation with a health professional as quickly as possible, Tánia contacted (around 8 a.m.) the public health services closest to her home. Due to the lack of availability of health personnel working on site and the overcrowding at the health care center, she was offered either an on-site consultation with a *specialist physician* but scheduled for 3 p.m. or a remote consultation (tele-consultation) scheduled for 10 a.m. (in a mobile truck parked in her neighborhood and equipped with video conferencing and medical diagnostic tools). Following this remote consultation, if necessary, the *specialist physician* will immediately send her an electronic medical prescription by telephone so that she can go to the pharmacy to get medications or a prescription to go for additional examinations at the nearest medical examination center. The price of the remote consultation would be the same as the price of the face-to-face consultation.

*If you were Tánia to what extend would you be willing to use the tele-consultation service proposed to her*?

Certainly would not o-----o-----o-----o-----o-----o-----o-----o-----o-----o-----o Certainly would

**Factor 1 + factor 5 variation and factor 1 second modality**

**Vignette 9**

Regina has not been feeling well for the last few days. The symptoms (fever, cold, cough, and diarrhea) indicate that she may have contracted an easily curable seasonal illness such as the flu or gastroenteritis. Having already seen the *generalist physician* the week before, Regina contacted him at 8 a.m. to have a follow-up consultation. Due to the lack of availability of health personnel working on site and the overcrowding at the health care center, she was offered either an on-site consultation with a *generalist physician* but scheduled for 3 p.m. or a remote consultation (tele-consultation) scheduled for 10 a.m. (in a mobile truck parked in her neighborhood and equipped with video conferencing and medical diagnostic tools). Following this remote consultation, if necessary, the *generalist physician* will immediately send her an electronic medical prescription by telephone so that she can go to the pharmacy to get medications or a prescription to go for additional examinations at the nearest medical examination center. The price of the remote consultation would be the same as the price of the face-to-face consultation.

*If you were Regina to what extend would you be willing to use the tele-consultation service proposed to her*?

Certainly would not o-----o-----o-----o-----o-----o-----o-----o-----o-----o-----o Certainly would

**Vignette 10**

Since Roberto learned two weeks ago that he had been selected for a job interview he has not been feeling well. The symptoms (slight increase in blood pressure, difficulty falling asleep, involuntary muscle contraction) indicate that he is experiencing an anxiety disorder. Having already seen the *generalist physician* the week before, Roberto contacted him at 8 a.m. to have a follow-up consultation. Due to the lack of availability of health personnel working on site and the overcrowding at the health care center, he was offered either an on-site consultation with a *generalist physician* but scheduled for 3 p.m. or a remote consultation (tele-consultation) scheduled for 10 a.m. (in a mobile truck parked in his neighborhood and equipped with video conferencing and medical diagnostic tools). Following this remote consultation, if necessary, the *generalist physician* will immediately send him an electronic medical prescription by telephone so that she can go to the pharmacy to get medications or a prescription to go for additional examinations at the nearest medical examination center. The price of the remote consultation would be the same as the price of the face-to-face consultation.

*If you were Roberto to what extend would you be willing to use the tele-consultation service proposed to him*?

Certainly would not o-----o-----o-----o-----o-----o-----o-----o-----o-----o-----o Certainly would

**---------------------------------------------------------------------------------------------------------------------------**

**Vignette 11**

For two weeks Paulo has not been feeling well. The symptoms (a loss of about 10kg in weight, gastroenteritis, skin lesions visible all over the body) indicate that he may have a serious disease (organic/infectious). Having already seen the *generalist physician* the week before, Paulo contacted him at 8 a.m. to have a follow-up consultation. Due to the lack of availability of health personnel working on site and the overcrowding at the health care center, he was offered either an on-site consultation with a *generalist physician* but scheduled for 3 p.m. or a remote consultation (tele-consultation) scheduled for 10 a.m. (in a mobile truck parked in his neighborhood and equipped with video conferencing and medical diagnostic tools). Following this remote consultation, if necessary, the *generalist physician* will immediately send him an electronic medical prescription by telephone so that she can go to the pharmacy to get medications or a prescription to go for additional examinations at the nearest medical examination center. The price of the remote consultation would be the same as the price of the face-to-face consultation.

*If you were Paulo to what extend would you be willing to use the tele-consultation service proposed to him*?

Certainly would not o-----o-----o-----o-----o-----o-----o-----o-----o-----o-----o Certainly would

**Vignette 12**

Since the death of her husband, two weeks ago, in a car accident, Natália is not feeling well. The symptoms (severe depression, multiple panic attacks, suicide attempt) indicate that she may be experiencing a very serious mental health problem. Having already seen the *generalist physician* the week before, Natália contacted him at 8 a.m. to have a follow-up consultation. Due to the lack of availability of health personnel working on site and the overcrowding at the health care center, she was offered either an on-site consultation with a *generalist physician* but scheduled for 3 p.m. or a remote consultation (tele-consultation) scheduled for 10 a.m. (in a mobile truck parked in her neighborhood and equipped with video conferencing and medical diagnostic tools). Following this remote consultation, if necessary, the *generalist physician* will immediately send her an electronic medical prescription by telephone so that she can go to the pharmacy to get medications or a prescription to go for additional examinations at the nearest medical examination center. The price of the remote consultation would be the same as the price of the face-to-face consultation.

*If you were Natália to what extend would you be willing to use the tele-consultation service proposed to her*?

Certainly would not o-----o-----o-----o-----o-----o-----o-----o-----o-----o-----o Certainly would

**Factor 1 + factor 5 variation and factor 1 second modality and factor 5 second modality**

**Vignette 13**

Flávia has not been feeling well for the last few days. The symptoms (fever, cold, cough, and diarrhea) indicate that she may have contracted an easily curable seasonal illness such as the flu or gastroenteritis. Having already seen the *specialist physician* the week before, Flávia contacted him at 8 a.m. to have a follow-up consultation. Due to the lack of availability of health personnel working on site and the overcrowding at the health care center, she was offered either an on-site consultation with a *specialist physician* but scheduled for 3 p.m. or a remote consultation (tele-consultation) scheduled for 10 a.m. (in a mobile truck parked in her neighborhood and equipped with video conferencing and medical diagnostic tools). Following this remote consultation, if necessary, the *specialist physician* will immediately send her an electronic medical prescription by telephone so that she can go to the pharmacy to get medications or a prescription to go for additional examinations at the nearest medical examination center. The price of the remote consultation would be the same as the price of the face-to-face consultation.

*If you were Flávia to what extend would you be willing to use the tele-consultation service proposed to her*?

Certainly would not o-----o-----o-----o-----o-----o-----o-----o-----o-----o-----o Certainly would

**Vignette 14**

Since Raul learned two weeks ago that he had been selected for a job interview he has not been feeling well. The symptoms (slight increase in blood pressure, difficulty falling asleep, involuntary muscle contraction) indicate that he is experiencing an anxiety disorder. Having already seen the *specialist physician* the week before, Raul contacted him at 8 a.m. to have a follow-up consultation. Due to the lack of availability of health personnel working on site and the overcrowding at the health care center, he was offered either an on-site consultation with a *specialist physician* but scheduled for 3 p.m. or a remote consultation (tele-consultation) scheduled for 10 a.m. (in a mobile truck parked in his neighborhood and equipped with video conferencing and medical diagnostic tools). Following this remote consultation, if necessary, the *specialist physician* will immediately send him an electronic medical prescription by telephone so that she can go to the pharmacy to get medications or a prescription to go for additional examinations at the nearest medical examination center. The price of the remote consultation would be the same as the price of the face-to-face consultation.

*If you were Raul to what extend would you be willing to use the tele-consultation service proposed to him*?

Certainly would not o-----o-----o-----o-----o-----o-----o-----o-----o-----o-----o Certainly would

**---------------------------------------------------------------------------------------------------------------------------**

**Vignette 15**

For two weeks Miguel has not been feeling well. The symptoms (a loss of about 10kg in weight, gastroenteritis, skin lesions visible all over the body) indicate that he may have a serious disease (organic/infectious). Having already seen the *specialist physician* the week before, Miguel contacted him at 8 a.m. to have a follow-up consultation. Due to the lack of availability of health personnel working on site and the overcrowding at the health care center, he was offered either an on-site consultation with a *specialist physician* but scheduled for 3 p.m. or a remote consultation (tele-consultation) scheduled for 10 a.m. (in a mobile truck parked in his neighborhood and equipped with video conferencing and medical diagnostic tools). Following this remote consultation, if necessary, the *specialist physician* will immediately send him an electronic medical prescription by telephone so that she can go to the pharmacy to get medications or a prescription to go for additional examinations at the nearest medical examination center. The price of the remote consultation would be the same as the price of the face-to-face consultation.

*If you were Miguel to what extend would you be willing to use the tele-consultation service proposed to him*?

Certainly would not o-----o-----o-----o-----o-----o-----o-----o-----o-----o-----o Certainly would

**Vignette 16**

Since the death of her husband, two weeks ago, in a car accident, Teresa is not feeling well. The symptoms (severe depression, multiple panic attacks, suicide attempt) indicate that she may be experiencing a very serious mental health problem. Having already seen the *specialist physician* the week before, Teresa contacted him at 8 a.m. to have a follow-up consultation. Due to the lack of availability of health personnel working on site and the overcrowding at the health care center, she was offered either an on-site consultation with a *specialist physician* but scheduled for 3 p.m. or a remote consultation (tele-consultation) scheduled for 10 a.m. (in a mobile truck parked in her neighborhood and equipped with video conferencing and medical diagnostic tools). Following this remote consultation, if necessary, the *specialist physician* will immediately send her an electronic medical prescription by telephone so that she can go to the pharmacy to get medications or a prescription to go for additional examinations at the nearest medical examination center. The price of the remote consultation would be the same as the price of the face-to-face consultation.

*If you were Teresa to what extend would you be willing to use the tele-consultation service proposed to her*?

Certainly would not o-----o-----o-----o-----o-----o-----o-----o-----o-----o-----o Certainly would

**Factor 1 + factor 5 variation, and factor 1 second modality, and factor 5 second modality, and factor 2 second modality**

**Vignette 17**

Helena has not been feeling well for the last few days. The symptoms (fever, cold, cough, and diarrhea) indicate that she may have contracted an easily curable seasonal illness such as the flu or gastroenteritis. With the goal of making an appointment to have a consultation with a health professional as quickly as possible, Helena contacted (around 8 a.m.) the public health services closest to her home. Due to the lack of availability of health personnel working on site and the overcrowding at the health care center, she was offered either an on-site consultation with a *generalist physician* but scheduled for 3 p.m. or a remote consultation (tele-consultation) scheduled for 10 a.m. (in a mobile truck parked in her neighborhood and equipped with video conferencing and medical diagnostic tools). Following this remote consultation, if necessary, the *generalist physician* will immediately send her an electronic medical prescription by telephone so that she can go to the pharmacy to get medications or a prescription to go for additional examinations at the nearest medical examination center. The price of the remote consultation would be 25% cheaper than the price of the face-to-face consultation.

*If you were Helena to what extend would you be willing to use the tele-consultation service proposed to her*?

Certainly would not o-----o-----o-----o-----o-----o-----o-----o-----o-----o-----o Certainly would

**Vignette 18**

Since Joaquim learned two weeks ago that he had been selected for a job interview he has not been feeling well. The symptoms (slight increase in blood pressure, difficulty falling asleep, involuntary muscle contraction) indicate that he is experiencing an anxiety disorder. With the goal of making an appointment to have a consultation with a health professional as quickly as possible, Joaquim contacted (around 8 a.m.) the public health services closest to his home. Due to the lack of availability of health personnel working on site and the overcrowding at the health care center, he was offered either an on-site consultation with a *generalist physician* but scheduled for 3 p.m. or a remote consultation (tele-consultation) scheduled for 10 a.m. (in a mobile truck parked in his neighborhood and equipped with video conferencing and medical diagnostic tools). Following this remote consultation, if necessary, the *generalist physician* will immediately send him an electronic medical prescription by telephone so that she can go to the pharmacy to get medications or a prescription to go for additional examinations at the nearest medical examination center. The price of the remote consultation would be 25% cheaper than the price of the face-to-face consultation.

*If you were Joaquim to what extend would you be willing to use the tele-consultation service proposed to him*?

Certainly would not o-----o-----o-----o-----o-----o-----o-----o-----o-----o-----o Certainly would

**---------------------------------------------------------------------------------------------------------------------------**

**Vignette 19**

For two weeks Tiago has not been feeling well. The symptoms (a loss of about 10kg in weight, gastroenteritis, skin lesions visible all over the body) indicate that he may have a serious disease (organic/infectious). With the goal of making an appointment to have a consultation with a health professional as quickly as possible, Tiago contacted (around 8 a.m.) the public health services closest to his home. Due to the lack of availability of health personnel working on site and the overcrowding at the health care center, he was offered either an on-site consultation with a *generalist physician* but scheduled for 3 p.m. or a remote consultation (tele-consultation) scheduled for 10 a.m. (in a mobile truck parked in his neighborhood and equipped with video conferencing and medical diagnostic tools). Following this remote consultation, if necessary, the *generalist physician* will immediately send him an electronic medical prescription by telephone so that she can go to the pharmacy to get medications or a prescription to go for additional examinations at the nearest medical examination center. The price of the remote consultation would be 25% cheaper than the price of the face-to-face consultation.

*If you were Tiago to what extend would you be willing to use the tele-consultation service proposed to him*?

Certainly would not o-----o-----o-----o-----o-----o-----o-----o-----o-----o-----o Certainly would

**Vignette 20**

Since the death of her husband, two weeks ago, in a car accident, Belona is not feeling well. The symptoms (severe depression, multiple panic attacks, suicide attempt) indicate that she may be experiencing a very serious mental health problem. With the goal of making an appointment to have a consultation with a health professional as quickly as possible, Belona contacted (around 8 a.m.) the public health services closest to her home. Due to the lack of availability of health personnel working on site and the overcrowding at the health care center, she was offered either an on-site consultation with a *generalist physician* but scheduled for 3 p.m. or a remote consultation (tele-consultation) scheduled for 10 a.m. (in a mobile truck parked in her neighborhood and equipped with video conferencing and medical diagnostic tools). Following this remote consultation, if necessary, the *generalist physician* will immediately send her an electronic medical prescription by telephone so that she can go to the pharmacy to get medications or a prescription to go for additional examinations at the nearest medical examination center. The price of the remote consultation would be 25% cheaper than the price of the face-to-face consultation.

*If you were Belona to what extend would you be willing to use the tele-consultation service proposed to her*?

Certainly would not o-----o-----o-----o-----o-----o-----o-----o-----o-----o-----o Certainly would

**Vignette 21**

Luisa has not been feeling well for the last few days. The symptoms (fever, cold, cough, and diarrhea) indicate that she may have contracted an easily curable seasonal illness such as the flu or gastroenteritis. With the goal of making an appointment to have a consultation with a health professional as quickly as possible, Luisa contacted (around 8 a.m.) the public health services closest to her home. Due to the lack of availability of health personnel working on site and the overcrowding at the health care center, she was offered either an on-site consultation with a *specialist physician* but scheduled for 3 p.m. or a remote consultation (tele-consultation) scheduled for 10 a.m. (in a mobile truck parked in her neighborhood and equipped with video conferencing and medical diagnostic tools). Following this remote consultation, if necessary, the *specialist physician* will immediately send her an electronic medical prescription by telephone so that she can go to the pharmacy to get medications or a prescription to go for additional examinations at the nearest medical examination center. The price of the remote consultation would be 25% cheaper than the price of the face-to-face consultation.

*If you were Luisa to what extend would you be willing to use the tele-consultation service proposed to her*?

Certainly would not o-----o-----o-----o-----o-----o-----o-----o-----o-----o-----o Certainly would

**Vignette 22**

Since Adelino learned two weeks ago that he had been selected for a job interview he has not been feeling well. The symptoms (slight increase in blood pressure, difficulty falling asleep, involuntary muscle contraction) indicate that he is experiencing an anxiety disorder. With the goal of making an appointment to have a consultation with a health professional as quickly as possible, Adelino contacted (around 8 a.m.) the public health services closest to his home. Due to the lack of availability of health personnel working on site and the overcrowding at the health care center, he was offered either an on-site consultation with a *specialist t physician* but scheduled for 3 p.m. or a remote consultation (tele-consultation) scheduled for 10 a.m. (in a mobile truck parked in his neighborhood and equipped with video conferencing and medical diagnostic tools). Following this remote consultation, if necessary, the *specialist physician* will immediately send him an electronic medical prescription by telephone so that she can go to the pharmacy to get medications or a prescription to go for additional examinations at the nearest medical examination center. The price of the remote consultation would be 25% cheaper than the price of the face-to-face consultation.

*If you were Adelino to what extend would you be willing to use the tele-consultation service proposed to him*?

Certainly would not o-----o-----o-----o-----o-----o-----o-----o-----o-----o-----o Certainly would

**---------------------------------------------------------------------------------------------------------------------------**

**Vignette 23**

For two weeks Justino has not been feeling well. The symptoms (a loss of about 10kg in weight, gastroenteritis, skin lesions visible all over the body) indicate that he may have a serious disease (organic/infectious). With the goal of making an appointment to have a consultation with a health professional as quickly as possible, Justino contacted (around 8 a.m.) the public health services closest to his home. Due to the lack of availability of health personnel working on site and the overcrowding at the health care center, he was offered either an on-site consultation with a *specialist physician* but scheduled for 3 p.m. or a remote consultation (tele-consultation) scheduled for 10 a.m. (in a mobile truck parked in his neighborhood and equipped with video conferencing and medical diagnostic tools). Following this remote consultation, if necessary, the *specialist physician* will immediately send him an electronic medical prescription by telephone so that she can go to the pharmacy to get medications or a prescription to go for additional examinations at the nearest medical examination center. The price of the remote consultation would be 25% cheaper than the price of the face-to-face consultation.

*If you were Justino to what extend would you be willing to use the tele-consultation service proposed to him*?

Certainly would not o-----o-----o-----o-----o-----o-----o-----o-----o-----o-----o Certainly would

**Vignette 24**

Since the death of her husband, two weeks ago, in a car accident, Aurora is not feeling well. The symptoms (severe depression, multiple panic attacks, suicide attempt) indicate that she may be experiencing a very serious mental health problem. With the goal of making an appointment to have a consultation with a health professional as quickly as possible, Aurora contacted (around 8 a.m.) the public health services closest to her home. Due to the lack of availability of health personnel working on site and the overcrowding at the health care center, she was offered either an on-site consultation with a *specialist physician* but scheduled for 3 p.m. or a remote consultation (tele-consultation) scheduled for 10 a.m. (in a mobile truck parked in her neighborhood and equipped with video conferencing and medical diagnostic tools). Following this remote consultation, if necessary, the *specialist physician* will immediately send her an electronic medical prescription by telephone so that she can go to the pharmacy to get medications or a prescription to go for additional examinations at the nearest medical examination center. The price of the remote consultation would be 25% cheaper than the price of the face-to-face consultation.

*If you were Aurora to what extend would you be willing to use the tele-consultation service proposed to her*?

Certainly would not o-----o-----o-----o-----o-----o-----o-----o-----o-----o-----o Certainly would

**Vignette 25**

Telma has not been feeling well for the last few days. The symptoms (fever, cold, cough, and diarrhea) indicate that she may have contracted an easily curable seasonal illness such as the flu or gastroenteritis. Having already seen the *generalist physician* the week before, Telma contacted him at 8 a.m. to have a follow-up consultation. Due to the lack of availability of health personnel working on site and the overcrowding at the health care center, she was offered either an on-site consultation with a *generalist physician* but scheduled for 3 p.m. or a remote consultation (tele-consultation) scheduled for 10 a.m. (in a mobile truck parked in her neighborhood and equipped with video conferencing and medical diagnostic tools). Following this remote consultation, if necessary, the *generalist physician* will immediately send her an electronic medical prescription by telephone so that she can go to the pharmacy to get medications or a prescription to go for additional examinations at the nearest medical examination center. The price of the remote consultation would be 25% cheaper than the price of the face-to-face consultation.

*If you were Telma to what extend would you be willing to use the tele-consultation service proposed to her*?

Certainly would not o-----o-----o-----o-----o-----o-----o-----o-----o-----o-----o Certainly would

**Vignette 26**

Since Augusto learned two weeks ago that he had been selected for a job interview he has not been feeling well. The symptoms (slight increase in blood pressure, difficulty falling asleep, involuntary muscle contraction) indicate that he is experiencing an anxiety disorder. Having already seen the *generalist physician* the week before, Augusto contacted him at 8 a.m. to have a follow-up consultation. Due to the lack of availability of health personnel working on site and the overcrowding at the health care center, he was offered either an on-site consultation with a *generalist physician* but scheduled for 3 p.m. or a remote consultation (tele-consultation) scheduled for 10 a.m. (in a mobile truck parked in his neighborhood and equipped with video conferencing and medical diagnostic tools). Following this remote consultation, if necessary, the *generalist physician* will immediately send him an electronic medical prescription by telephone so that she can go to the pharmacy to get medications or a prescription to go for additional examinations at the nearest medical examination center. The price of the remote consultation would be 25% cheaper than the price of the face-to-face consultation.

*If you were Augusto to what extend would you be willing to use the tele-consultation service proposed to him*?

Certainly would not o-----o-----o-----o-----o-----o-----o-----o-----o-----o-----o Certainly would

**---------------------------------------------------------------------------------------------------------------------------**

**Vignette 27**

For two weeks Carlos has not been feeling well. The symptoms (a loss of about 10kg in weight, gastroenteritis, skin lesions visible all over the body) indicate that he may have a serious disease (organic/infectious). Having already seen the *generalist physician* the week before, Carlos contacted him at 8 a.m. to have a follow-up consultation. Due to the lack of availability of health personnel working on site and the overcrowding at the health care center, he was offered either an on-site consultation with a *generalist physician* but scheduled for 3 p.m. or a remote consultation (tele-consultation) scheduled for 10 a.m. (in a mobile truck parked in his neighborhood and equipped with video conferencing and medical diagnostic tools). Following this remote consultation, if necessary, the *generalist physician* will immediately send him an electronic medical prescription by telephone so that she can go to the pharmacy to get medications or a prescription to go for additional examinations at the nearest medical examination center. The price of the remote consultation would be 25% cheaper than the price of the face-to-face consultation.

*If you were Carlos to what extend would you be willing to use the tele-consultation service proposed to him*?

Certainly would not o-----o-----o-----o-----o-----o-----o-----o-----o-----o-----o Certainly would

**Vignette 28**

Since the death of her husband, two weeks ago, in a car accident, Farida is not feeling well. The symptoms (severe depression, multiple panic attacks, suicide attempt) indicate that she may be experiencing a very serious mental health problem. Having already seen the *generalist physician* the week before, Farida contacted him at 8 a.m. to have a follow-up consultation. Due to the lack of availability of health personnel working on site and the overcrowding at the health care center, she was offered either an on-site consultation with a *generalist physician* but scheduled for 3 p.m. or a remote consultation (tele-consultation) scheduled for 10 a.m. (in a mobile truck parked in her neighborhood and equipped with video conferencing and medical diagnostic tools). Following this remote consultation, if necessary, the *generalist physician* will immediately send her an electronic medical prescription by telephone so that she can go to the pharmacy to get medications or a prescription to go for additional examinations at the nearest medical examination center. The price of the remote consultation would be 25% cheaper than the price of the face-to-face consultation.

*If you were Farida to what extend would you be willing to use the tele-consultation service proposed to her*?

Certainly would not o-----o-----o-----o-----o-----o-----o-----o-----o-----o-----o Certainly would

**Factor 1 + factor 5 variation and factor 1 second modality and factor 5 second modality**

**Vignette 29**

Leticia has not been feeling well for the last few days. The symptoms (fever, cold, cough, and diarrhea) indicate that she may have contracted an easily curable seasonal illness such as the flu or gastroenteritis. Having already seen the *specialist physician* the week before, Leticia contacted him at 8 a.m. to have a follow-up consultation. Due to the lack of availability of health personnel working on site and the overcrowding at the health care center, she was offered either an on-site consultation with a *specialist physician* but scheduled for 3 p.m. or a remote consultation (tele-consultation) scheduled for 10 a.m. (in a mobile truck parked in her neighborhood and equipped with video conferencing and medical diagnostic tools). Following this remote consultation, if necessary, the *specialist physician* will immediately send her an electronic medical prescription by telephone so that she can go to the pharmacy to get medications or a prescription to go for additional examinations at the nearest medical examination center. The price of the remote consultation would be 25% cheaper than the price of the face-to-face consultation.

*If you were Leticia to what extend would you be willing to use the tele-consultation service proposed to her*?

Certainly would not o-----o-----o-----o-----o-----o-----o-----o-----o-----o-----o Certainly would

**Vignette 30**

Since Ismael learned two weeks ago that he had been selected for a job interview he has not been feeling well. The symptoms (slight increase in blood pressure, difficulty falling asleep, involuntary muscle contraction) indicate that he is experiencing an anxiety disorder. Having already seen the *specialist physician* the week before, Ismael contacted him at 8 a.m. to have a follow-up consultation. Due to the lack of availability of health personnel working on site and the overcrowding at the health care center, he was offered either an on-site consultation with a *specialist physician* but scheduled for 3 p.m. or a remote consultation (tele-consultation) scheduled for 10 a.m. (in a mobile truck parked in his neighborhood and equipped with video conferencing and medical diagnostic tools). Following this remote consultation, if necessary, the *specialist physician* will immediately send him an electronic medical prescription by telephone so that she can go to the pharmacy to get medications or a prescription to go for additional examinations at the nearest medical examination center. The price of the remote consultation would be 25% cheaper than the price of the face-to-face consultation.

*If you were Ismael to what extend would you be willing to use the tele-consultation service proposed to him*?

Certainly would not o-----o-----o-----o-----o-----o-----o-----o-----o-----o-----o Certainly would

**---------------------------------------------------------------------------------------------------------------------------**

**Vignette 31**

For two weeks Tiago has not been feeling well. The symptoms (a loss of about 10kg in weight, gastroenteritis, skin lesions visible all over the body) indicate that he may have a serious disease (organic/infectious). Having already seen the *specialist physician* the week before, Tiago contacted him at 8 a.m. to have a follow-up consultation. Due to the lack of availability of health personnel working on site and the overcrowding at the health care center, he was offered either an on-site consultation with a *specialist physician* but scheduled for 3 p.m. or a remote consultation (tele-consultation) scheduled for 10 a.m. (in a mobile truck parked in his neighborhood and equipped with video conferencing and medical diagnostic tools). Following this remote consultation, if necessary, the *specialist physician* will immediately send him an electronic medical prescription by telephone so that she can go to the pharmacy to get medications or a prescription to go for additional examinations at the nearest medical examination center. The price of the remote consultation would be 25% cheaper than the price of the face-to-face consultation.

*If you were Tiago to what extend would you be willing to use the tele-consultation service proposed to him*?

Certainly would not o-----o-----o-----o-----o-----o-----o-----o-----o-----o-----o Certainly would

**Vignette 32**

Since the death of her husband, two weeks ago, in a car accident, Amélia is not feeling well. The symptoms (severe depression, multiple panic attacks, suicide attempt) indicate that she may be experiencing a very serious mental health problem. Having already seen the *specialist physician* the week before, Amélia contacted him at 8 a.m. to have a follow-up consultation. Due to the lack of availability of health personnel working on site and the overcrowding at the health care center, she was offered either an on-site consultation with a *specialist physician* but scheduled for 3 p.m. or a remote consultation (tele-consultation) scheduled for 10 a.m. (in a mobile truck parked in her neighborhood and equipped with video conferencing and medical diagnostic tools). Following this remote consultation, if necessary, the *specialist physician* will immediately send her an electronic medical prescription by telephone so that she can go to the pharmacy to get medications or a prescription to go for additional examinations at the nearest medical examination center. The price of the remote consultation would be 25% cheaper than the price of the face-to-face consultation.

*If you were Amélia to what extend would you be willing to use the tele-consultation service proposed to her*?

Certainly would not o-----o-----o-----o-----o-----o-----o-----o-----o-----o-----o Certainly would

1. **RECEIVED STRESS SCALE (**Cohen & Williamson [31])

INSTRUCTIONS:

You will be asked various questions below. They are about your feelings and thoughts during the past month. Each time, we ask you to rate how you felt in the past month.

Although some questions are similar, there are differences between them, and each should be seen as independent from the others. The best way to do this is to respond quickly. Do not try to count the number of times you have felt this or that way, but rather indicate the answers that seem closest to reality from the five choices offered:

1: Never.

2: Almost never (rarely).

3: Sometimes.

4: Quite often.

5: Very often.

Put a cross in the box corresponding to your choice.

| In the past month how many times | Never  1 | Almost never  2 | Sametimes  3 | Quite often  4 | Very often  5 |
| --- | --- | --- | --- | --- | --- |
| 1… have you been disturbed by an unexpected event? |  |  |  |  |  |
| 2… Did you find it difficult to control the important things in your life? |  |  |  |  |  |
| 3… have you felt nervous and stressed? |  |  |  |  |  |
| 4… have you successfully faced the small daily problems and annoyances? |  |  |  |  |  |
| 5… have you felt that you are coping effectively with the important changes that are occurring in your life? |  |  |  |  |  |
| 6… Did you feel confident in your ability to deal with your personal issues? |  |  |  |  |  |
| 7… did you feel that things were going the way you wanted? |  |  |  |  |  |
| 8… have you thought that you cannot take on all the things you have to do? |  |  |  |  |  |
| 9… were you able to control your nervousness? |  |  |  |  |  |
| 10… did you feel like you were in control? |  |  |  |  |  |
| 11… Did you feel irritated because the events were beyond your control? |  |  |  |  |  |
| 12… Did you find that the difficulties accumulated so much that you could not control them? |  |  |  |  |  |

Note: items 4, 5, 6, 7, 9, 10 are noted in reverse

1. **SOCIO-DEMOGRAPHIC CHARACTERISTICS**

Age ______

Sex______

Education level (in terms of number of years of study counting from the first year of primary school) _______

Socio-economic status (the family monthly income) _______

Religious practitioner: Yes ___ Non___

Religion name _______

1. Please note that the original data collection material is written in Portuguese. This is an English translation version. [↑](#footnote-ref-1)
